# Binding of *Candida albicans* to Human CEACAM1 and CEACAM6 Modulates the Inflammatory Response of Intestinal Epithelial Cells

**DOI:** 10.1128/mBio.02142-16

**Published:** 2017-03-14

**Authors:** Esther Klaile, Mario M. Müller, Miriam R. Schäfer, Ann-Katrin Clauder, Sabina Feer, Kerstin A. Heyl, Magdalena Stock, Tilman E. Klassert, Peter F. Zipfel, Bernhard B. Singer, Hortense Slevogt

**Affiliations:** a Septomics Research Center, Jena University Hospital, Jena, Germany; b Center for Sepsis Control and Care (CSCC), University Hospital Jena, Jena, Germany; c Department of Infection Biology, Leibniz Institute for Natural Product Research and Infection Biology, Jena, Germany; d Faculty of Biology and Pharmacy, Infection Biology, Friedrich Schiller University, Jena, Germany; e Institute of Anatomy, Medical Faculty, University Duisburg-Essen, Essen, Germany; University of Texas Health Science Center

**Keywords:** C2BBe1, CEA, CEACAM1, CEACAM3, CEACAM5, CEACAM6, *Candida albicans*, *Candida glabrata*, carcinoembryonic antigen-related cell adhesion molecule, epithelial cells, innate immunity

## Abstract

*Candida albicans* colonizes human mucosa, including the gastrointestinal tract, as a commensal. In immunocompromised patients, *C. albicans* can breach the intestinal epithelial barrier and cause fatal invasive infections. Carcinoembryonic antigen-related cell adhesion molecule 1 (CEACAM1; CD66a), CEACAM5 (CEA), and CEACAM6 (CD66c) are immunomodulatory receptors expressed on human mucosa and are recruited by bacterial and viral pathogens. Here we show for the first time that a fungal pathogen (i.e., *C. albicans*) also binds directly to the extracellular domain of human CEACAM1, CEACAM3, CEACAM5, and CEACAM6. Binding was specific for human CEACAMs and mediated by the N-terminal IgV-like domain. In enterocytic C2BBe1 cells, *C. albicans* caused a transient tyrosine phosphorylation of CEACAM1 and induced higher expression of membrane-bound CEACAM1 and soluble CEACAM6. Lack of the CEACAM1 receptor after short hairpin RNA (shRNA) knockdown abolished CXCL8 (interleukin-8) secretion by C2BBe1 cells in response to *C. albicans*. In CEACAM1-competent cells, the addition of recombinant soluble CEACAM6 reduced the *C. albicans*-induced CXCL8 secretion.

## IMPORTANCE

The present study demonstrates for the first time that fungal pathogens can be recognized by at least four members of the immunomodulatory CEACAM receptor family: CEACAM1, -3, -5, and -6. Three of the four receptors (i.e., CEACAM1, -5, and -6) are expressed in mucosal cells of the intestinal tract, where they are implicated in immunomodulation and control of tissue homeostasis. Importantly, the interaction of the major fungal pathogen in humans *Candida albicans* with CEACAM1 and CEACAM6 resulted in an altered epithelial immune response. With respect to the broad impact of CEACAM receptors on various aspects of the innate and the adaptive immune responses, in particular epithelial, neutrophil, and T cell behavior, understanding the role of CEACAMs in the host response to fungal pathogens might help to improve management of superficial and systemic fungal infections.

## INTRODUCTION

*Candida* species colonize human mucosal surfaces as commensals but can turn to pathogenic behavior in immunocompromised patients. *Candida albicans* and *Candida glabrata* cause oral, esophageal, vaginal, or urinary mucosal infections as well as fatal invasive infections with a high crude and attributable mortality (1). The majority of systemic infections are caused by endogenous *C. albicans* strains colonizing the patient’s gastrointestinal tract.

To reach the bloodstream, *C. albicans* has to breach the intestinal epithelial barrier, which separates the gut lumen with its microbiota from the host organism (1). Virulence mechanisms and factors employed by this polymorphic yeast include adhesion via adhesins followed by active penetration of the hyphal growth form and the degradation of epithelial cell junction proteins via proteolysis by secreted aspartyl proteinases (2).

The interaction of *C. albicans* with mucosal epithelial cells lining the intestinal tract and local immune cells determines whether homeostasis will be maintained or if the detection will initiate an inflammatory response (3). Mucosal epithelial cells express a variety of fungal pattern recognition receptors (PRRs) to initiate and orchestrate immune responses (4, 5), but the exact composition of the receptors employed by enteric epithelial cells to recognize *C. albicans* is currently unknown.

Receptors of the carcinoembryonic antigen-related cell adhesion molecule (CEACAM) family are widely expressed on immune cells (6) and epithelial cells of the respiratory and the gastrointestinal tract (7, 8). CEACAM1 (CD66a, Bgp) has the broadest expression range and is found on epithelial and endothelial cells as well as on leukocytes (6). Its major isoforms encompass a transmembrane domain and either a short or a long cytoplasmic domain, the latter bearing two immunoreceptor tyrosine-based inhibition motifs (ITIMs) responsible for the propagation of CEACAM1-dependent signaling (9). CEACAM3 (CD66d, CGM1) is granulocyte specific, and the major isoform possesses a transmembrane domain and an intracellular immunoreceptor tyrosine-based activation motif (ITAM) (10). CEACAM5 (CEA, CD66e), found on epithelia, and CEACAM6 (CD66c, NCA), expressed on epithelia and granulocytes, both have glycosylphosphatidylinositol (GPI) anchors (11). All four CEACAMs are highly glycosylated (12) and affect basic cellular functions like proliferation and apoptosis/survival and are able to regulate immune functions (6, 13).

In the human intestine, CEACAM1, -5, -6, and -7 are expressed on mucosal epithelial cells (8). CEACAM1, CEACAM5, and CEACAM6 are receptors for a variety of bacterial pathogens and mediate adhesion and internalization—e.g., of *Neisseria gonorrhoeae*, *Neisseria meningitidis*, different *Escherichia coli* strains, *Moraxella catarrhalis*, *Haemophilus influenzae*, *Salmonella* species, and *Helicobacter pylori* (11, 14). Many of these bacteria have evolved different, structurally unrelated surface proteins that all target the human-specific extracellular immunoglobulin V (IgV)-like amino-terminal domain of CEACAMs (11).

The mucosal immune reaction is comprised of immune cell and epithelial cell interactions and responses (3), many of which can be regulated by CEACAMs. Probably best studied is the function of CEACAM1 as a comodulatory receptor on T cells (15), where the ligation of CEACAM1 with soluble agonists represses CD3-mediated T cell responses like lymphokine secretion and cytolytic functions. On neutrophils, the ligation of CEACAMs leads to their activation (10, 16, 17), and we could show that CEACAM1 also mediates the delay of apoptosis (18). In human pulmonary epithelial cells, binding of *M. catarrhalis* to CEACAM1 not only mediates bacterial adhesion (19, 20) but also attenuates Toll-like receptor 2 (TLR2)-mediated immune responses (19, 21).

In the present study, we identified four members of the human CEACAM family as novel *C. albicans* receptors and characterize CEACAM1 as an important immunoregulatory fungal receptor on intestinal epithelial cells.

## RESULTS

### Extracellular domains of human CEACAM receptors bind directly to *C. albicans.*

**FIG 1:**
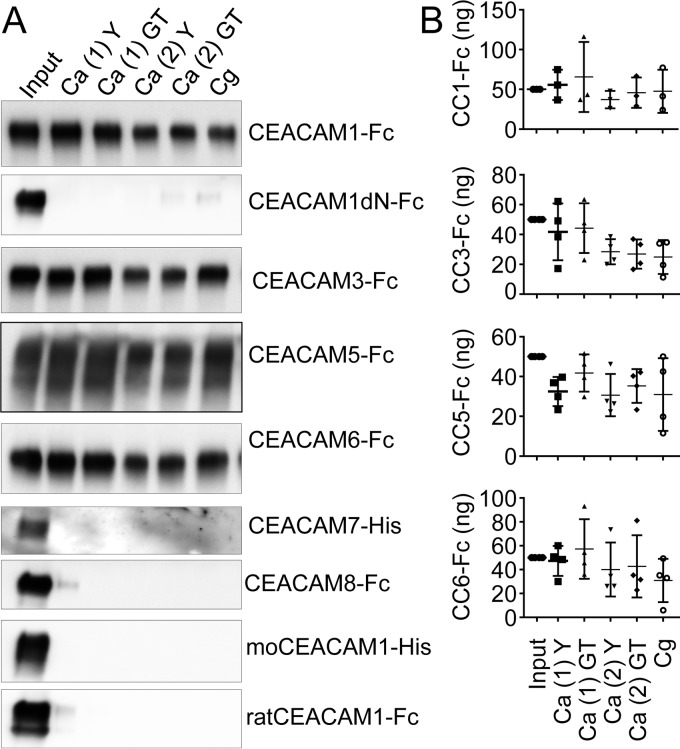
Recombinant CEACAM receptors bind to *Candida* species. (A and B) For pulldown assays, yeast cells (Y) and germ tubes (GT) of *C. albicans* SC5314 [Ca (1)] or *C. albicans* C28a [Ca (2)] and *C. glabrata* 2001 (Cg) yeast cells were incubated with 1 µg of the indicated recombinant proteins. Fusion proteins bound to *Candida* surfaces were analyzed by Western blotting (A). moCEACAM1-His, mouse CEACAM1-His. As a positive control, 5% of the input (50 ng) was loaded in the first lane, and the amounts of precipitated CEACAM1-Fc, CEACAM3-Fc, CEACAM5-Fc, and CEACAM6-Fc were quantified (B). Statistical analysis was performed with the two-sided unpaired *t* test. Bars in all graphs depict the mean (wide bars) ± SD (narrow bars, if applicable).

In order to test for specific binding of recombinant CEACAM extracellular domains to fungal pathogens, we performed pulldown assays with yeast cells and germ tubes of two different *C. albicans* strains (SC5314 and C28a) and *C. glabrata* yeast cells (strain 2001) (Fig. 1A and B). Four recombinant proteins encompassing the extracellular domains of human CEACAM1, CEACAM3, CEACAM5, or CEACAM6 bound to the tested *Candida* species recognizing both *C. albicans* growth forms (i.e., yeast cells and germ tubes). Recombinant proteins comprising human CEACAM8 or CEACAM7 extracellular domains did not show specific binding to fungal cells.

The binding was specific for the human CEACAM1 extracellular domain, since the homologous fusion proteins of mouse CEACAM1 and rat CEACAM1 extracellular domains were not precipitated by either *Candida* strain (Fig. 1). We tested the dependence of the human CEACAM1-*Candida* interaction on the availability of the N-terminal IgV-like domain, which mediates the majority of CEACAM-pathogen interactions, by using a recombinant human CEACAM1-Fc protein lacking the respective domain (CEACAM1deltaN-Fc [here, CEACAM1dN-Fc]) in the pulldown assay. The absence of the N-terminal CEACAM1 IgV-like domain completely abrogated binding to the *C. albicans* strains and also to *C. glabrata* (Fig. 1).

**FIG 2:**
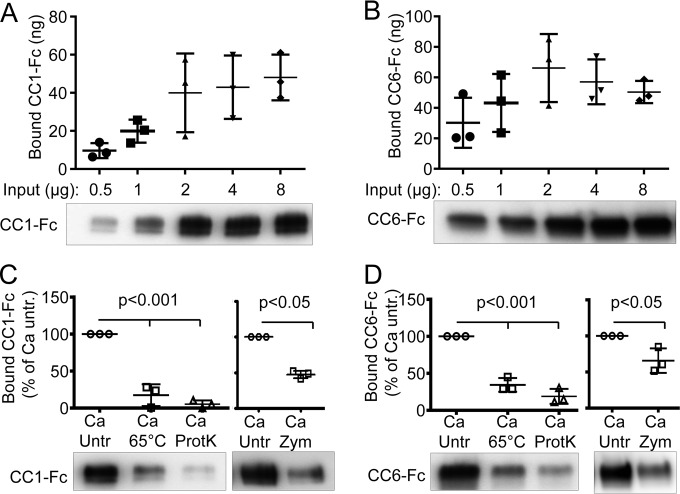
Analysis of CEACAM1 and CEACAM6 binding to *C. albicans*. (A and B) *C. albicans* SC5314 yeast cells were incubated with the indicated amounts of CEACAM1-Fc (CC1-Fc) (A) or CEACAM6-Fc (CC6-Fc) (B). Fusion proteins bound to *Candida* surfaces were analyzed by Western blotting (lower panels). Bands were quantified, and total amounts of bound CEACAM1 (A) and CEACAM6 (B) are shown in the graphs. (C and D) *C. albicans* SC5314 yeast cells were left untreated (Untr) or were pretreated for 1 h at 65°C or with 7.5 µg proteinase K (ProtK) at 37°C or with Zymolyase (Zym) at room temperature before pulldown assays with 1 µg CEACAM1-Fc (C) or CEACAM6-Fc (D) were performed. Amounts of bound CEACAM1 (C) and CEACAM6 (D) were quantified: the respective relative amounts compared to those in untreated *Candida* cells (100%) are shown in the graphs. Statistical analysis was performed with the two-sided paired *t* test. Bars in all graphs depict the mean (wide bars) ± SD (narrow bars, if applicable).

Since *C. albicans* SC5314 displayed the highest binding toward recombinant CEACAM molecules, this strain was used for further analysis. We next assessed whether binding of CEACAM1-Fc and CEACAM6-Fc could be saturated in pulldown assays (Fig. 2A and B). When using 0.5, 1, or 2 µg Fc fusion protein per 2 × 10^8^ yeast cells, the quantity of bound CEACAM1-Fc and CEACAM6-Fc increased with larger amounts of fusion proteins. The addition of 4 or 8 µg of recombinant Fc fusion proteins did not result in further enhancement of the bound fraction of either CEACAM1-Fc or CEACAM6-Fc, revealing that the binding of both recombinant fusion proteins with cell surface structures found on the cell walls of *C. albicans* was indeed saturable.

### The interaction of extracellular CEACAM domains with *C. albicans* requires intact cell wall proteins.

In order to analyze whether the binding of CEACAM proteins to *Candida* cells was mediated by cell wall mannan/glucans, by proteins, or by protein-bound glycans, pulldowns were performed with either heat-denatured or proteolytically digested (proteinase K) *C. albicans* yeast cells. Precipitation of both CEACAM1-Fc and CEACAM6-Fc was strongly reduced when *C. albicans* yeast cells were pretreated for 1 h at 65°C or with proteinase K (Fig. 2C and D), indicating that the interaction is mediated by a proteinaceous, heat-labile component. Deglycosylation of yeast cells using Zymolyase also reduced the binding of both proteins significantly. However, deglycosylation also released large amounts of cell wall proteins (see Fig. S5 in the supplemental material), thus preventing any clear conclusions with regard to the impact of carbohydrates on the CEACAM-*Candida* interaction.

### *C. albicans* increases the expression of CEACAM molecules in intestinal epithelial cells.

**FIG 3:**
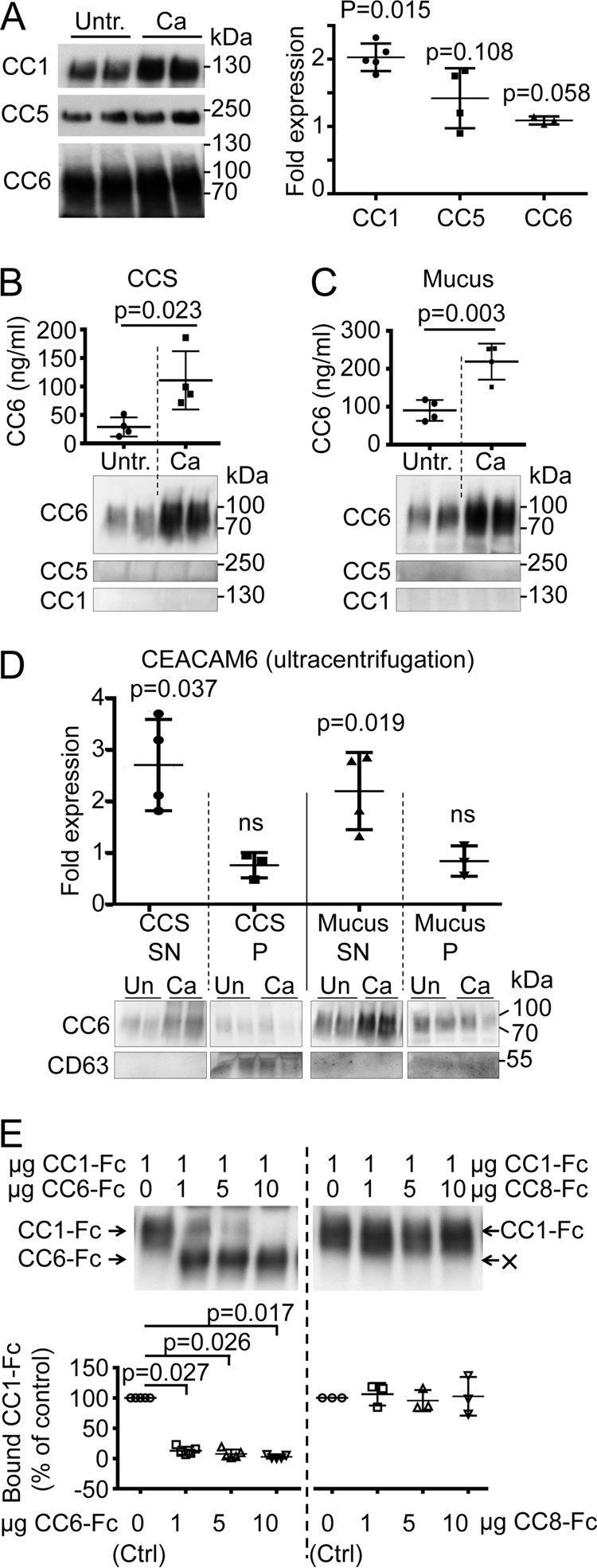
*C. albicans* induces CEACAM expression in C2BBe1 cells. C2BBe1 cells were left untreated (Untr.) or were stimulated with UV-inactivated *C. albicans* SC5314 yeast cells (Ca) for 72 h in duplicate. Cell culture supernatants (CCS) were carefully removed, and cells were washed twice to collect the mucus fraction before cell lysis. (A) Cell lysates were analyzed by Western blotting for CEACAM1 (CC1; *n =* 5), CEACAM5 (CC5; *n =* 4), and CEACAM6 (CC6; *n =* 4) expression (left panels). CEACAM bands were quantified: the respective mean fold changes from duplicates after *C. albicans* treatment are shown in the graph (right panel). (B and C) Cell culture supernatants (B) and mucus fractions (C) were analyzed by Western blotting for CEACAM1 (*n =* 3), CEACAM5 (*n =* 3), and CEACAM6 (*n =* 4) expression (lower panels). CEACAM6 bands were quantified: the mean CEACAM6 concentrations from duplicates are presented in the graphs (B and C, upper panels). (D) Analysis of soluble and extracellular vesicle (EV)-bound CEACAM6 in CCS and mucus fractions by ultracentrifugation. Supernatants (SN) and pellets (P) of the indicated samples after centrifugation at 100,000 × *g* for 2-h were analyzed by Western blotting (lower panels) for the presence of CEACAM6 and CD63 (positive control, EV marker). CEACAM6 bands were quantified, and mean fold changes from the duplicates of four independent experiments after *C. albicans* treatment are given in the graph (upper panels). Un, untreated. (E) *C. albicans* SC5314 yeast cells were incubated with 1 µg CEACAM1-Fc in the presence of different amounts of CEACAM6-Fc (*n* = 5) or CEACAM8-Fc (*n* = 3) as indicated, and samples were analyzed by Western blotting for the presence of the respective recombinant proteins (upper panels). Note the absence of CEACAM8-Fc in the precipitates (“X,” upper right panel). CEACAM1 bands were quantified: the relative amounts of bound CEACAM1 compared to CEACAM1-Fc alone (100%) are shown in the graphs (lower panels). Statistical analysis was performed with the two-sided paired *t* test for panels A, D, and E and the two-sided unpaired *t* test for panels B and C. Bars in all graphs depict the mean (wide bars) ± SD (narrow bars, if applicable). ns, not significant.

Since bacterial CEACAM-binding pathogens increase the expression of their own CEACAM receptors in epithelial cells (7), we tested whether *C. albicans* interactions with human intestinal epithelial C2BBe1 cells also affected expression levels of epithelial CEACAM receptors (Fig. 3). C2BBe1 cells form tight monolayers and can be differentiated by prolonged cultivation to express several morphological and functional characteristics of the mature enterocyte (22). One easily traceable feature during differentiation of C2BBe1 cells is the “dome formation” that C2BBe1 cells start to develop a few days after reaching confluence (see Fig. S1A in the supplemental material).

10.1128/mBio.02142-16.1 FIG S1  CEACAM receptors are expressed differentiation-dependent in C2BBe1 cells. (A) Micrographs of C2BBe1 cells cultivated for 14 days after reaching confluence. Both panels show the same area: the left panel is focused on the monolayer on the surface of the cell culture plate, and the right panel is focused on the tip of the “dome.” Scale bars, 100 µm. (B) Expression of transcripts of CEACAM5, CEACAM6, and CEACAM7 (with no discrimination between the two isoforms) and of the four major human CEACAM1 isoforms (CC1-4L, CC1-4S, CC1-3L, and CC1-3S) was determined by qPCR analysis using specific primer pairs (7) in untreated C2BBe1 cells differentiated for 21 days in cell culture plates. Peptidylprolyl isomerase B (PPIB) and hypoxanthine phosphoribosyltransferase 1 (HPRT) were used as controls. RNA extraction, reverse transcription, primer design, qPCR, and data analysis were done as described elsewhere (7). Cycle thresholds (CT) of three independent experiments are shown in the graph. Note that the long CEACAM1 isoforms (CC1-4L and CC1-3L) and CEACAM7 display the lowest mRNA expression levels. (C) C2BBe1 cells were cultured on cell culture plates or Transwell filters for 7 or 21 days as indicated. Cell lysates were analyzed by Western blotting for the expression of CEACAM1, CEACAM5, CEACAM6, CEACAM7, and actin. As positive controls, cells were treated for 48 h with 100 ng/ml IFN-γ (IFNg) in order to induce enhanced CEACAM expression. Note the reduced CEACAM1 and CEACAM6 expression and the abolished CEACAM5 expression in well-differentiated cells. Panels are representative of at least two independent experiments. Untr., untreated. (D) C2BBe1 cells were cultured on cell culture plates for 14 days. Cells were analyzed by flow cytometry for the expression of CEACAM1, CEACAM5, CEACAM6, and CEACAM7. As positive controls, cells were treated for 48 h with 100 ng/ml interferon gamma or with 1 mM H_2_O_2_ (two stimulations at 0 h and at 24 h) to induce enhanced CEACAM expression. Note that, as already shown for the parental Caco-2 cells (24), interferon gamma did not alter the CEACAM7 expression in C2BBe1 cells (C and D). CEACAM7 was only detected after stimulation with 1 mM H_2_O_2_. Panels are representative of at least two independent experiments. Download FIG S1, PDF file, 1.7 MB. Copyright © 2017 Klaile et al. 2017 Klaile et al. This content is distributed under the terms of the Creative Commons Attribution 4.0 International license.

Similar to their parental Caco-2 cell line and other epithelial cell lines (23, 24), C2BBe1 cells expressed various amounts of CEACAM1, CEACAM5, and CEACAM6, depending on the state of differentiation (Fig. S1). When kept subconfluent, all three CEACAM receptors were expressed at a high level, as judged by quantitative PCR (qPCR) (Fig. S1B) and Western blotting (Fig. S1C). When differentiated for 14 to 21 days on cell culture plates, C2BBe1 cells displayed reduced CEACAM1 expression, minimal CEACAM5 expression, and mildly decreased CEACAM6 expression (Fig. 3A; Fig. S1C). C2BBe1 cells differentiated for 21 days on Transwell filters displayed no detectable CEACAM5 expression and a strongly reduced CEACAM1 and CEACAM6 expression. C2BBe1 cells in all stages of differentiation showed a similar ability to respond to interferon gamma (IFN-γ) treatment, with an increase in CEACAM1, CEACAM5, and CEACAM6 expression levels (Fig. S1C). qPCR analysis revealed CEACAM7 mRNA in untreated C2BBe1 cells (Fig. S1B), but no protein was detectable by Western blotting (Fig. S1C) or flow cytometry (Fig. S1D) in untreated or in IFN-γ-treated cells. For further experiments, 21-day-differentiated cells grown on cell culture plates were used if not stated otherwise.

Incubation of C2BBe1 cells with UV-inactivated *C. albicans* (CaUV) for 72 h affected the expression of both membrane-bound and also soluble CEACAMs (Fig. 3). Immunoblotting revealed a significant 2-fold increase in CEACAM1 expression in whole-cell lysates (Fig. 3A). After 72 h, cell culture supernatants (CCS) and mucus fractions of untreated C2BBe1 cells contained large amounts of soluble CEACAM6 (12 to 51 ng/ml) (Fig. 3B and C) but no soluble CEACAM1 or CEACAM5 (Fig. 3B and C, lower panels). Stimulation with CaUV for 72 h induced a strong increase in CEACAM6 release into the CCS (2-fold) and the mucus fractions (8-fold) with CEACAM6 concentrations of 61 to 121 ng/ml (Fig. 3B and C).

In order to determine whether CEACAM6 found in CCS and mucus fractions was soluble or extracellular vesicle (EV) bound, we performed ultracentrifugation experiments (Fig. 3D). Supernatants and pellets obtained after ultracentrifugation were analyzed by Western blotting for the presence of CEACAM6, and CD63, an EV marker protein used here as a positive control for successful EV pelleting. CEACAM6 in CCS and mucus fractions was found mainly in the soluble supernatant after ultracentrifugation, although some minor portion of CEACAM6 was also pelleted and detected in the EV-containing, CD63-positive fractions. Interestingly, the CaUV-induced increase of released CEACAM6 was only detectable in the soluble fractions after ultracentrifugation and not in the pellet fractions. Together with the apparent molecular mass of CEACAM6 bands detected by immunoblotting in whole-cell lysates and CCS/mucus fractions of 70 to 100 kDa (Fig. 3A, B, and C), this demonstrates that the CaUV-induced CEACAM6 protein is a highly glycosylated (12), soluble variant comprising all three Ig domains no longer GPI anchored to EV.

### Increasing amounts of CEACAM6 prevent CEACAM1 binding to *C. albicans* cell surface epitopes.

CEACAM6 (25) and CEACAM8 (26) can both bind to the extracellular IgV-like domain of CEACAM1. In addition, CEACAM6 but not CEACAM8 demonstrated binding to *Candida* cell surface components, as shown in Fig. 1. Since soluble as well as cell-bound CEACAM6 was present at a high excess compared to CEACAM1 in C2BBe1 cells, and a soluble isoform of CEACAM6 was upregulated by CaUV treatment, we next tested whether the presence of soluble CEACAM6 affects the binding of CEACAM1 to cell surface components present on *C. albicans* yeast cells. In competitive pulldown assays, *C. albicans* cells were incubated with CEACAM1 in the absence or presence of increasing amounts of CEACAM6-Fc or CEACAM8-Fc, respectively (Fig. 3E). Recombinant CEACAM8, the non-*Candida*-binding control fusion protein, had no effect on the quantity of precipitated recombinant CEACAM1 even at high ratios. In contrast, the addition of CEACAM6 strikingly decreased the amount of precipitated CEACAM1-Fc. Already at a 1:1 ratio, CEACAM6-Fc diminished CEACAM1-Fc binding to *Candida* surfaces by almost 90%. Higher ratios augmented this effect, and at a 1:10 ratio, hardly any CEACAM1-Fc bound to *Candida* cell surfaces was detectable by immunoblotting.

### CEACAM expression does not influence *C. albicans* adhesion to epithelial cells.

**FIG 4:**
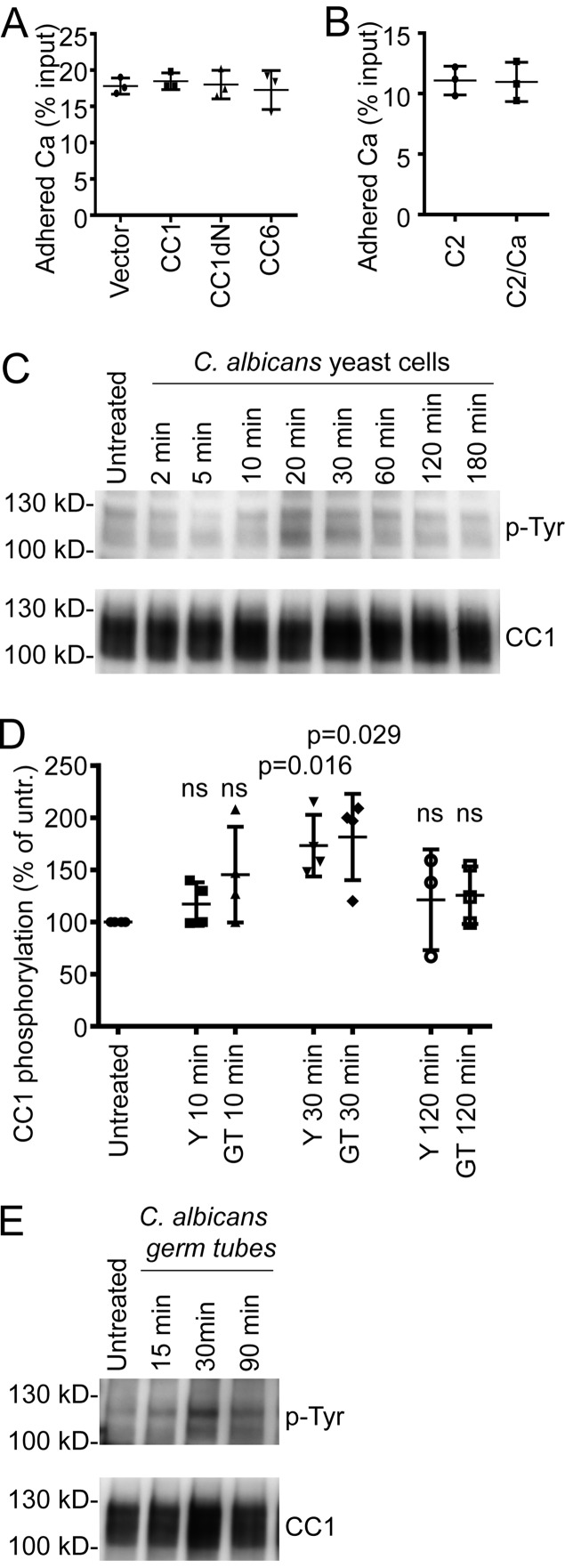
CEACAM1 becomes phosphorylated by *C. albicans* but does not affect yeast cell adhesion to epithelial monolayers. (A) HeLa cells stably transfected with empty vector, human CEACAM1-4L (CC1), a human CEACAM1-4L deletion mutant lacking the N-terminal IgV-like domain (CC1dN), or human CEACAM6 (CC6) were incubated with *C. albicans* yeast cells for 60 min. Shown are mean adhered cells as a percentage of input from three individual experiments done in triplicate. (B) For adhesion assays, C2BBe1 cells were carefully washed to remove mucus from the cell monolayer. *C. albicans* yeast cells were preincubated with cell culture supernatants conditioned by C2BBe1 cells left untreated (C2) or treated with UV-inactivated *C. albicans* yeast cells for 72 h (C2/Ca) and were allowed to adhere to C2BBe1 cells for 30 min in the presence of the respective preconditioned media. Shown are mean adhered cells as a percentage of input from three individual experiments performed in triplicate. (C and D) To analyze the tyrosine phosphorylation of CEACAM1, C2BBe1 cells were incubated with *C. albicans* SC5314 yeast cells for the indicated times (*n =* 4). Cells were lysed, CEACAM1 was immunoprecipitated, and samples were analyzed by Western blotting for phosphotyrosine and subsequently for CEACAM1 (C). Note that both CEACAM1-4L (upper p-Tyr band) and CEACAM-3L (lower p-Tyr band) isoforms become phosphorylated. (D) Phosphotyrosine signals were quantified: the fold changes after *C. albicans* treatment compared to untreated cells (100%) are shown in the graph. (E) C2BBe1 cells were incubated with *C. albicans* germ tubes and analyzed as described for panel C. Statistical analysis was performed with the two-sided paired *t* test for panels A and B and the two-sided unpaired *t* test for panel D. Bars in all graphs depict the mean (wide bars) ± SD (narrow bars, if applicable). ns, not significant.

To test whether CEACAM1 or CEACAM6 expression on epithelial surfaces affects the adhesion of *C. albicans* to epithelial monolayers, we allowed *C. albicans* yeast cells to adhere to HeLa cells transfected with either CEACAM1-4L, CEACAM6, or the non-*Candida* binding CEACAM1 mutant CEACAM1-4L-deltaN (here CEACAM1-4LdN) (Fig. 4A). Adhering *Candida* cells were plated, and CFU were counted, revealing that overexpression of either CEACAM in HeLa cells did not affect *Candida* adhesion to epithelial surfaces (Fig. 4A).

To examine if the increased amounts of soluble CEACAM6 found in cell culture supernatants of CaUV-treated C2BBe1 cells had any effect on the *C. albicans* adhesion to C2BB1e surfaces, we tested the adhesion of yeast cells in the presence of medium conditioned by untreated or CaUV-treated C2BBe1 cells (Fig. 4B). Plating of adhering *Candida* cells revealed that under both conditions, equal numbers of *C. albicans* CFU were recovered.

### *C. albicans* stimulation of C2BBe1 cells induces a transient CEACAM1 tyrosine phosphorylation.

Although we did not find any consequences of CEACAM molecule expression on *Candida* adhesion to epithelial surfaces, we studied whether binding of *C. albicans* to extracellular domains of CEACAM molecules on epithelial cells would evoke an activation of CEACAM1, i.e., the phosphorylation of tyrosine residues within the immunoreceptor tyrosine-based inhibition motif (ITIM) of the long CEACAM1 isoforms (CEACAM1-4L and CEACAM1-3L, summarily termed CEACAM1-L). C2BBe1 cells were either left untreated or were incubated with live *C. albicans* yeast cells for the indicated times and lysed. Immunoprecipitated CEACAM1 was then tested for tyrosine phosphorylation by Western blotting (Fig. 4C and D). Upon *Candida* yeast cell stimulation, CEACAM1-4L and CEACAM1-3L displayed a transient tyrosine phosphorylation (note the two distinct p-Tyr bands in Fig. 4C) that peaked between 20 and 30 min and afterward declined (Fig. 4C and D). A similar transient increase in CEACAM1–l-tyrosine phosphorylation was detected when *C. albicans* germ tubes were used for C2BB1e cell stimulations (Fig. 4E).

### CEACAM1 knockdown in C2BBe1 cells.

**FIG 5:**
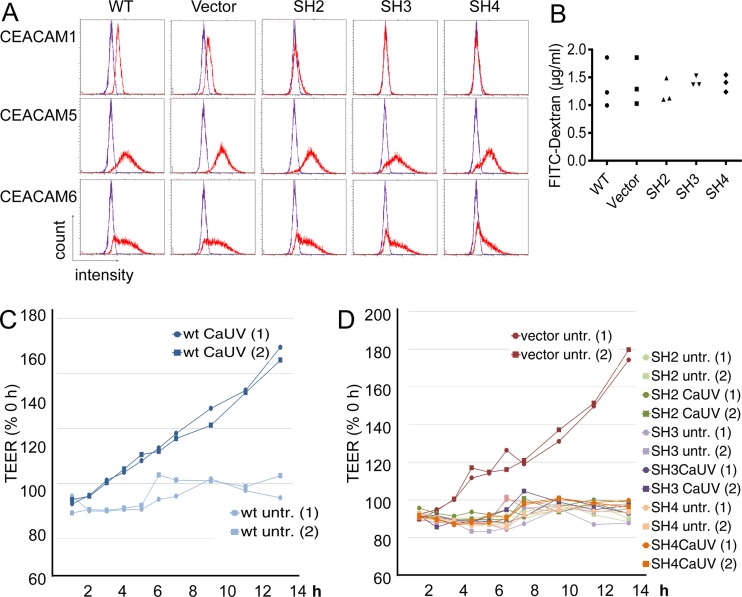
CEACAM1 regulates alterations in the transepithelial electrical resistance of C2BBe1 cells in response to *C. albicans*. C2BBe1 cells were transfected with the control vector and three different shRNA vectors targeting CEACAM1 (SH2, SH3, and SH4). (CEACAM1-negative populations were sorted.) (A) Analysis of CEACAM1, CEACAM5, and CEACAM6 cell surface expression of the wild-type, vector control, and SH2, SH3, and SH4 cell lines by flow cytometry. Red lines indicate CEACAM-specific antibody, and violet lines indicate the IgG1 control antibody. (B) All cell lines were grown on Transwell filters and tested for differences in permeability by the addition of FITC-dextran to the upper chamber. After 24 h, the concentration of FITC-dextran in the medium from the lower chamber was analyzed in a fluorescence reader. The graph shows the FITC-dextran concentrations of triplicate wells from one representative experiment out of three. (C and D) Wild-type (wt) cells (C) and transfected cell lines (D) were grown on Transwell filters, and transepithelial electric resistance (TEER) was measured in untreated (untr.) cells and cells stimulated with UV-inactivated *C. albicans* SC5314 yeast cells. Relative TEER values are shown as the percentage of the value at 0 h of each well. The graphs display measurements of duplicate wells [(1) and (2), respectively] from one representative experiment out of three.

In order to address a possible function of CEACAM1 activation in the C2BBe1 response to *C. albicans*, three short hairpin RNA (shRNA)-mediated CEACAM1 knockdown cell lines (SH2, SH3, and SH4) were generated. Wild-type (WT) cells, vector control cells, and cells of the three CEACAM1 shRNA-mediated lines SH2, SH3, and SH4 were analyzed for CEACAM1, CEACAM5, and CEACAM6 expression (Fig. 5A). Flow cytometry revealed that the SH2 cells retained a minimal CEACAM1 expression on their cell surface while SH3 and SH4 cells did not show detectable CEACAM1 cell surface expression. All cells displayed comparable CEACAM5 and CEACAM6 levels on their surface (Fig. 5A). Western blot analysis of cell lysates gave similar results, revealing a minimal CEACAM1 expression in SH2 cells and nearly undetectable CEACAM1 expression levels in SH3 and SH4 cells (see Fig. S2 in the supplemental material).

**FIG 6:**
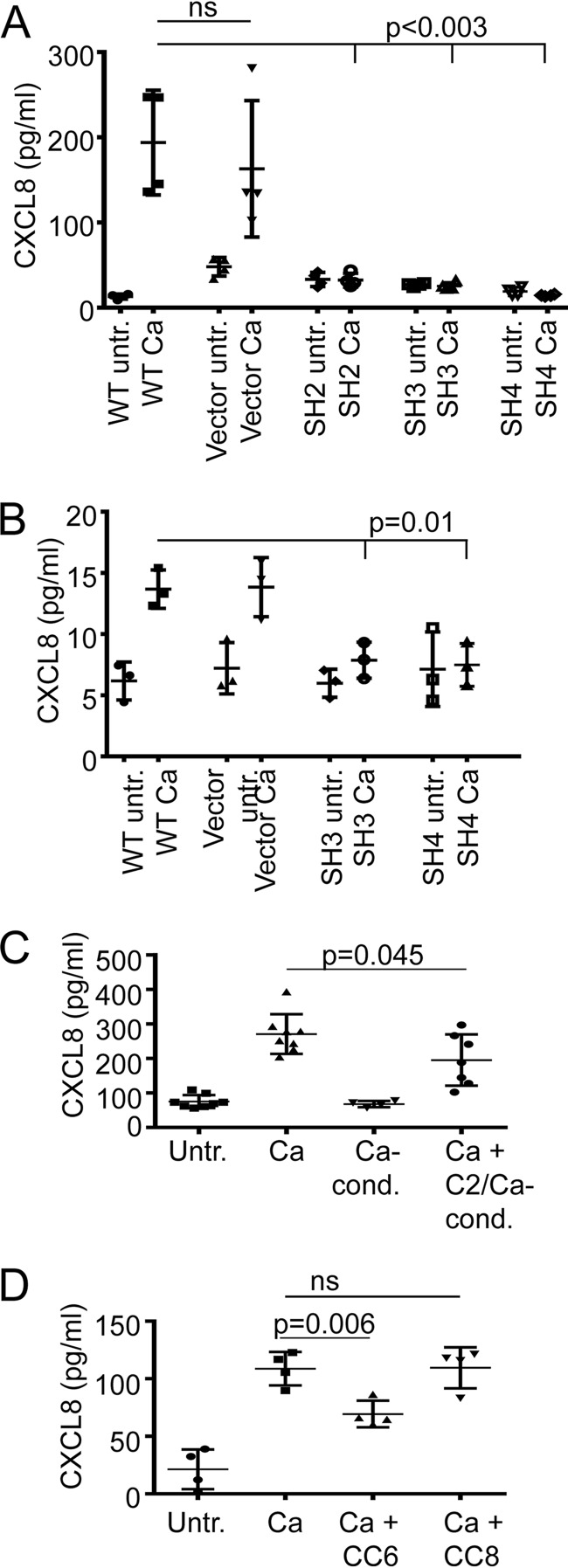
CEACAM1 and CEACAM6 regulate the CXCL8 release of C2BBe1 cells in response to *C. albicans*. (A) The C2BBe1 wild-type, vector control, and SH2, SH3, and SH4 cell lines were grown in cell culture plates and either left untreated (untr.) or were incubated with UV-inactivated *C. albicans* SC5314 yeast cells (Ca; *n =* 4). Supernatants were harvested after 72 h and tested for CXCL8 concentrations by ELISA. (B) The C2BBe1 wild-type, vector control, and SH3 and SH4 cell lines were grown on Transwell filters and either left untreated or incubated apically with UV-inactivated *C. albicans* SC5314 yeast cells (*n =* 3). Medium from the lower chambers was harvested after 72 h and tested for CXCL8 concentrations by ELISA. (C) C2BBe1 cells were either left untreated (*n =* 8) or were treated with UV-inactivated *C. albicans* SC5314 yeast cells (Ca; *n =* 8), or treated with medium conditioned by live *C. albicans* SC5314 cells (Ca-Cond; *n =* 4). C2BBe1 cells were also treated with UV-inactivated *C. albicans* SC5314 yeast cells preincubated in medium conditioned by C2BBe1 cells stimulated with UV-inactivated *C. albicans* SC5314 yeast cells (Ca + C2/Ca-Cond; *n =* 7). Supernatants were harvested after 96 h and tested for CXCL8 concentrations by ELISA. (D) To test the influence of recombinant CEACAM6 on the CXCL8 induction by *C. albicans*, C2BBe1 cells were either left untreated, treated with UV-inactivated *C. albicans* SC5314 yeast cells (Ca), or treated with UV-inactivated *C. albicans* SC5314 yeast cells in the presence of 30 µg/ml CEACAM6-Fc (Ca + CC6) or CEACAM8-Fc (Ca + CC8). Supernatants were harvested after 52 h and tested for CXCL8 concentrations by ELISA. Bars in all graphs depict the mean (wide bars) ± SD (narrow bars, if applicable). Statistical analysis was performed with the two-sided unpaired *t* test. ns, not significant.

10.1128/mBio.02142-16.2 FIG S2  Expression of CEACAM receptors in C2BBe1 cells transfected with CEACAM1 shRNA. C2BBe1 cells were transfected with CEACAM1 shRNA vectors and sorted for CEACAM1-negative cells (Fig. 6A). Cell lysates were analyzed by Western blotting for the expression of CEACAM1 (CC1), CEACAM5 (CC5), CEACAM6 (CC6), and TOM-20 (mitochondrial outer membrane protein, loading control). Download FIG S2, PDF file, 0.7 MB. Copyright © 2017 Klaile et al. 2017 Klaile et al. This content is distributed under the terms of the Creative Commons Attribution 4.0 International license.

### CEACAM1 controls *C. albicans*-induced alterations in the TEER.

Since *C. albicans* has the capacity of traversing the intestinal epithelial barrier and can also alter its permeability (27), and CEACAM1 expression also influences vascular (28, 29) and colonic (30) permeability, we next tested whether CEACAM1 knockdown affects the epithelial barrier function. All cell lines were grown on Transwell filters and tested for two permeability parameters: i.e., the ability to prevent the permeation of fluorescein isothiocyanate (FITC)-dextran and transepithelial electrical resistance (TEER).

With regard to paracellular FITC-dextran translocation across unstimulated monolayers, no differences were found in CEACAM1 knockdown cells (Fig. 5B). Also, no differences in the TEER of monolayers of untreated cells were found between all five cell lines tested. TEER values reached 280 to 500 Ω/cm^2^ after 7 days of confluence, 350 to 700 Ω/cm^2^ after 14 days of confluence, and 850 to 1,300 Ω/cm^2^ after 21 days of confluence. C2BBe1 wild-type cells (Fig. 5C) and vector-transfected cells (Fig. 5D) reacted to the stimulation with CaUV with a considerable increase in TEER. In contrast, when monolayers of SH2, SH3, and SH4 CEACAM1 knockdown cells were incubated with CaUV, no significant alterations of TEER values was detected (Fig. 5D).

When wild-type, vector, and SH3 CEACAM1 knockdown cells were challenged with live *C. albicans*, no differences in TEER values were found between the three cell lines (see Fig. S3 in the supplemental material). After 6 h, the increasing damage of the monolayers resulted in a steady decline of TEER values. Together, these results demonstrate that CEACAM1 expressed on epithelial cells can modulate cellular TEER responses evoked by *Candida* adhesion to epithelial surfaces, but CEACAM1 expression does not affect epithelial penetration and barrier breakdown by live *C. albicans* cells.

10.1128/mBio.02142-16.3 FIG S3  Reduction in transepithelial electric resistance (TEER) induced by live *C. albicans* cells. C2BBe1 wild-type cells (wt Ca), vector control-transfected [Vector (Ca)], or CEACAM1-SH3 vector-transfected [Sh3 Ca)] cells were grown on Transwell filters for 21 days, and TEER was measured in cells stimulated with live *C. albicans* cells (MOI, 100). Relative TEER is shown as a percentage of the value at 0 h of each well. The graphs display the means of measurements of duplicate wells from one representative experiment out of two. Download FIG S3, PDF file, 0.7 MB. Copyright © 2017 Klaile et al. 2017 Klaile et al. This content is distributed under the terms of the Creative Commons Attribution 4.0 International license.

### CEACAM1 is essential for *C. albicans*-induced CXCL8 release.

Next, we wondered if CEACAM1 expression and its recognition by *C. albicans* affected the epithelial immune response after fungal detection. Therefore, we evaluated the CXCL8 production induced by CaUV in the different C2BBe1 cell lines. In wild-type and vector control cells, CaUV treatment lead to the release of CXCL8 into the cell culture supernatant (Fig. 6A). Importantly, lack of CEACAM1 in SH2, SH3, and SH4 cells completely abolished CXCL8 secretion. In order to test whether the CEACAM1 knockdown cells were still able to secrete CXCL8 independently of a *Candida*-CEACAM1 interaction, all cell lines were stimulated with a cytokine mix (interleukin-1 [IL-1β], tumor necrosis factor alpha [TNF-α], and IFN-γ). The wild-type, vector, and the three knockdown cell lines released a similar amount of CXCL8 after stimulation with the proinflammatory cytokine cocktail (see Fig. S4 in the supplemental material). Since C2BBe1 cells differentiated on Transwell filters display a strongly reduced CEACAM1 expression, we tested the different cell lines also under these conditions for CXCL8 release after *C. albicans* challenge. Again, only wild-type and vector control cells, but none of the CEACAM1 knockdown cell lines, displayed CXCL8 secretion induced by CaUV (Fig. 6B). Interestingly, even taking into account that due to technical reasons 4-fold less cells per milliliter of medium were analyzed, the CaUV-induced amount of CXCL8 was reduced in Transwell differentiated cells compared to wild-type and vector control cells grown in tissue culture plates (12 to 15 and 136 to 247 pg/ml, respectively), likely due to the reduced CEACAM1 expression of well-differentiated C2BBe1 cells (Fig. S1C).

10.1128/mBio.02142-16.4 FIG S4  Cytomix-induced CXCL8 induction in C2BBe1 cells and transfectants. C2BBe1 wild-type cells (wt), vector-transfected cells (vector), and shRNA vector-transfected cells (SH2, SH3, and SH4) were either left untreated (untr) or were incubated with cytomix (cyto [25 ng/ml IL-1β, 50 ng/ml TNF-α, and 50 ng/ml IFN-γ]). Supernatants were harvested after 48 h and tested for CXCL8 concentrations by ELISA. Mean concentrations of triplicate wells from one representative experiment out of two are shown. Download FIG S4, PDF file, 0.6 MB. Copyright © 2017 Klaile et al. 2017 Klaile et al. This content is distributed under the terms of the Creative Commons Attribution 4.0 International license.

10.1128/mBio.02142-16.5 FIG S5  Deglycosylation releases proteins from the *Candida albicans* cell wall. Five hundred microliters of wet *Candida albicans* pellets was solved in 1 ml PBS and either left untreated (Untr) or incubated with 3 mg of Zymolyase (Zym [AMS Biotechnology, Abington, United Kingdom]) for 1 h at room temperature. Yeast cells were removed by centrifugation at 3,000 × *g* for 2 min, and supernatants were further cleared by centrifugation at 16,000 × *g* for 10 min at 4°C. Cleared supernatants were heated to 99°C for 8 min under reducing conditions (5× reducing sample buffer, consisting of 14.5% [wt/vol] SDS, 0.3 M Tris-HCl [pH 6.8], 50% glycerol, 0.015% [wt/vol] bromphenol blue, and 20 mM DTT). Fifteen microliters was run on 15-well Mini-Protean TGX gels (4 to 15%) and either transferred to nitrocellulose membranes for Western blot analysis using a polyclonal rabbit anti-*Candida albicans* antibody (BP1006; Acris Antibodies Germany, Herford, Germany) (A) or subjected to Coomassie brilliant blue staining (B) and subsequent silver staining (C). Lane M in each panel contains molecular mass markers. Download FIG S5, PDF file, 1.9 MB. Copyright © 2017 Klaile et al. 2017 Klaile et al. This content is distributed under the terms of the Creative Commons Attribution 4.0 International license.

### *C. albicans* induction of CXCL8 production requires adhesion to C2BBe1 cells.

To test if the CXCL8 response of C2BB1e cells was evoked by a soluble factor secreted by *C. albicans* or due to the fungal interaction with epithelial surface proteins, we tested CXCL8 secretion in response to *C. albicans* SC5314-conditioned complete growth medium (Dulbecco’s modified Eagle’s medium [DMEM]–20% fetal bovine serum [FBS]), but no induction of CXCL8 release was found (Fig. 6C).

Interestingly, when we incubated C2BBe1 cells with CaUV in the presence of medium conditioned by CaUV-challenged C2BBe1 cells, we found a significant reduction in CXCL8 secretion by almost 30% (Fig. 6C), indicating that a soluble factor released by C2BBe1 cells into the cell culture medium decreased the proinflammatory response of C2BB1e cells toward *C. albicans*. since (i) soluble CEACAM6 was found in increasing amounts in cell culture supernatants after CaUV treatment (Fig. 3B and D), (ii) soluble CEACAM6 was interfering with the binding of CEACAM1 to *C. albicans* cell surface components (Fig. 3E), and (iii) CEACAM1 was essential to elicit CXCL8 secretion in C2BB1e cells in response to *C. albicans* (Fig. 6A and B), we next tested whether the presence of soluble recombinant CEACAM6-Fc affected the CXCL8 secretion of C2BB1e cells treated with CaUV. Indeed, we found that CaUV stimulation in the presence of soluble recombinant CEACAM6-Fc protein decreased the proinflammatory CXCL8 secretion of C2BB1e cells by 36% (Fig. 6D). Control incubations with CEACAM8-Fc did not influence the CXCL8 release.

## DISCUSSION

Here, we show for the first time, that the fungal pathogens *C. albicans* and *C. glabrata* bind to recombinant human CEACAM1, CEACAM3, CEACAM5, and CEACAM6. Similar to many bacterial pathogens, binding was restricted to members of the human CEACAM family and was mediated primarily by the N-terminal IgV-like domain (11), as we demonstrated for CEACAM1. The interaction is likely mediated by a proteinaceous component of the yeasts, since heat inactivation and proteinase K treatment of *C. albicans* as well as the release of cell wall proteins by deglycosylation strongly reduced the precipitation of recombinant CEACAM1 and CEACAM6. This is not surprising since all interactions of CEACAM receptors with pathogens published so far are mediated by specific bacterial or viral surface proteins (6).

Mucosal epithelial cells express CEACAM6 in three different forms: soluble or membrane-bound by a GPI anchor either on the cell surface or on extracellular vesicles (EVs) (31). All forms are able to regulate various aspects of immunity since CEACAM6 can engage in homophilic and heterophilic interactions with pathogens and host receptors like CEACAM1, CEACAM5, CEACAM8, hnRNP, DC-SIGN, β1 integrin, and β3 integrin (32).

Importantly, recombinant CEACAM6 protein was able to prevent the bulk of recombinant CEACAM1 protein binding to *C. albicans* in pulldown assays already at equal amounts. Recombinant CEACAM8 had no influence on CEACAM1 binding to *C. albicans*. Recombinant CEACAM8, like CEACAM6, is able to bind to CEACAM1 (21), but in contrast to CEACAM6, it is not able to bind to *Candida* cells. While we cannot exclude the possibility that direct binding of CEACAM6 to CEACAM1 is responsible for the effect of CEACAM6 on CEACAM1-*Candida* interaction, these results rather suggest that CEACAM1 and CEACAM6 share the same binding epitope on *C. albicans* and that CEACAM6 binds with a higher affinity than CEACAM1.

The “decoration” of *C. albicans* (and possibly also other pathogens [11]) with soluble CEACAM6 could alter the host reaction toward the organism by preventing its ligation of CEACAM1 and impeding the CEACAM1-mediated host response: e.g., the CEACAM1-dependent CXCL8 release described in this study. Since humans excrete up to 70 mg of CEACAM6 and CEACAM5 per day in their feces (33), such a scenario is not unlikely and might either be a host strategy to help maintain homeostasis in the intestinal mucosa, or it could be an immune evasion strategy adopted by the pathogen.

In parallel to the induction of soluble CEACAM6, *C. albicans* strongly increased the expression of membrane-bound CEACAM1 in intestinal epithelial cells. In contrast to findings with bacterial pathogens (11), ectopic expression of cell-bound CEACAM1 and CEACAM6 did not lead to enhanced binding of *C. albicans* to epithelial cells. This indicates that with regard to the fungal pathogen *C. albicans*, immunomodulatory rather than adhesive functions of the CEACAM receptors are of primary importance.

It is particularly interesting that the *C. albicans*-induced CXCL8 release and the transepithelial resistance of intestinal epithelial C2BBe1 cells (i.e., proinflammatory responses) depended on the presence of CEACAM1. While this finding is in agreement with our recent discovery that the interaction of *Helicobacter pylori* with CEACAM1 results in CXCL8 release in gastric epithelial cells (14), it is in contrast to studies describing inhibitory functions of CEACAM1 in human pulmonary epithelial cells, where ligation of CEACAM1 by *M. catarrhalis* and *N. meningitidis* leads to a reduced immune response to TLR2 agonists, resulting in the attenuation of inflammatory responses and allowing for immunoevasion strategies (19, 21). Taken together, these studies indicate that CEACAM1 belongs to a small group of ITIM-bearing immune receptors that can induce both pro- and anti-inflammatory signaling (34). It can thus have different functions in the gastrointestinal mucosa and in the respiratory tract, or it may react in a discriminative way to different stimuli/pathogens. Since CEACAM1 executes its immunomodulatory functions mostly via the regulation of the activity of other immune receptors (9, 19, 21), the expression and signaling properties of these receptors in the respective tissues can also influence the final outcome of CEACAM1 signaling. Whether the *Candida*-induced tyrosine phosphorylation of CEACAM1 is of importance for the CXCL8 release or the increase in transepithelial electrical resistance remains to be elucidated. Also, a more detailed investigation of the effect of cell-bound CEACAM6 on the enterocytic response to fungal pathogens will be of importance, since it also influences intestinal barrier functions and host susceptibility to CEACAM6-recognizing *E. coli* (35).

In the human intestinal mucosa, CXCL8 release of epithelial cells as found in this study will likely result in the recruitment of neutrophils, which are the first line of defense when coping with fungal infections (3) and play an important role in preventing *C. albicans* dissemination from the gut (36). Since the ligation of CEACAM1 to pathogens (9) or soluble agonists (37) can activate neutrophils or prolong the neutrophil life span (18), the presence of soluble CEACAM6 could also influence the neutrophil response to *C. albicans*. Soluble CEACAM6 might either prevent CEACAM1-mediated pathogen recognition by saturating the CEACAM-recognizing epitope(s) on the yeast, or it could promote neutrophil activation by direct binding to CEACAM1. Similarly, since CEACAM1 also regulates functions of various other immune cell types, including activated T and B cells, the CEACAM-*Candida* interactions can likely influence a broad spectrum of immune responses (9, 38). However, further *in vitro* and *in vivo* studies are needed to address the functions of CEACAM receptors in the response of the different immune cell types to *C. albicans*.

The identification of four novel human *C. albicans* receptors by the present study adds crucial information to the challenge of elucidating the host response to this yeast, which is the basis for a better management of *Candida* infections. We propose that CEACAM receptors are implicated in the maintenance of the homeostasis between the host mucosa and the colonizing yeast. Soluble CEACAM6 can control the fungal interaction with CEACAM1 on the cell surface, and only when *Candida* cells bind CEACAM1 (e.g., due to mucosal damage) do the epithelial cells respond in a proinflammatory fashion—i.e., tightening the epithelial barrier and attracting neutrophils by CXCL8 secretion.

## MATERIALS AND METHODS

### Materials and antibodies.

All materials used were from Sigma-Aldrich Chemie GmbH (Steinheim, Germany) or Merck-Millipore (Merck Chemicals GmbH, Darmstadt, Germany) unless stated otherwise. The following mouse monoclonal antibodies were used: anti-human CEACAM1, B3-17 (flow cytometry) and C5-1X (Western blot); anti-human CEACAM6, 1H7-4B; anti-human CEACAM5, 308-3-3; pan-human CEACAM1/3/4/5/6/8, 6G5J (all from B. B. Singer, Essen, Germany); anti-human CEACAM7, BAC2 (Santa Cruz Biotechnology, Inc., Heidelberg, Germany), antiphosphotyrosine, 4G10 (Millipore, Merck Chemicals GmbH, Darmstadt, Germany); and mouse IgG1 isotype control antibody (Antibodies-online GmbH, Aachen, Germany). The following polyclonal antibodies were used: phycoerythrin (PE)-conjugated goat anti-mouse IgG H+L (Antibodies-online GmbH, Aachen, Germany), horseradish peroxidase (HRP)-coupled goat anti-mouse IgG and HRP-coupled goat anti-rabbit IgG (both Dianova, Hamburg, Germany), and rabbit anti-CEA IgG (pan-human CEACAM; Dako Deutschland GmbH, Hamburg, Germany).

### Cell lines and fungal strains.

C2BBe1 cells were purchased from the American Type Culture Collection (ATCC [Manassas, VA]) and propagated in a mixture of DMEM, 20% FBS, and penicillin-streptomycin (all from Invitrogen, Darmstadt, Germany) in BD collagen I (BioCoat) 6-well plates/flasks or in 12-well Transwell inserts (polycarbonate, 3-µm pore) (all VWR International GmbH, Darmstadt, Germany). For experiments, cells were seeded at 80% confluence (plates) or 100% confluence (Transwell inserts) and differentiated for 20 to 24 days if not stated otherwise.

In order to generate CEACAM1 knockdown cell lines, C2BBe1 cells were transfected with control vector or with shRNA vectors (SureSilencing shRNA plasmid for human CEACAM1, KH00037N; SA Biosciences/Qiagen, Hilden, Germany) using Lipofectamine LTX and Plus reagent (Life Technologies, Inc., GmbH, Darmstadt, Germany). Transfected cells were selected for up to 10 days in culture medium containing 1 mg/ml of Geneticin; thereafter, cells were selected in 0.5 mg/ml Geneticin (Invitrogen, Darmstadt, Germany). Cells were stained for CEACAM1 (B3-17 PE-conjugated secondary antibody), and CEACAM1-negative cells were sorted by flow cytometry on a FACS Aria II instrument (Becton, Dickinson GmbH, Heidelberg, Germany). Three populations with a strongly reduced CEACAM1 expression were obtained with three different shRNA sequences, respectively: C2BBe1-SH2, C2BBe1-SH3, and C2BBe1-SH4 (Fig. 5A; Fig. S3).

HeLa cells were purchased from the Leibniz Institute DSMZ-German Collection of Microorganisms and Cell Cultures (Braunschweig, Germany) and propagated in RPMI with 10% FBS and penicillin-streptomycin. Cells were transfected with pRC/CMV vectors containing the cDNA of human CEACAM1-4L, human CEACAM1-4L-dN (lacking the N-terminal IgV-like domain), human CEACAM6, or empty vector (kindly provided by Wolfgang Zimmermann, University Hospital, Munich, Germany) (39). Cell surface expression of CEACAM proteins was confirmed by flow cytometry (see Fig. S6 in the supplemental material).

10.1128/mBio.02142-16.6 FIG S6  CEACAM cell surface expression in transfected HeLa cells. HeLa cells were transfected with vectors encoding (A) human CEACAM1-4L (CEACAM1), (B) human CEACAM1-4L lacking the N-terminal IgG-like domain (CEACAM1dN), (C) or human CEACAM6 or (D) empty vector. CEACAMs were detected by flow cytometry following indirect immunofluorescence staining with the following antibodies and corresponding IgG controls: (A) B3/17 (anti-CEACAM1), (B) polyclonal pan-CEA (anti-CEACAM1/3/4/5/6/8), (C) 1H7-4B (anti-CEACAM6), and (D) 6G5J (anti-CEACAM1/3/4/5/6/8). Download FIG S6, PDF file, 1.2 MB. Copyright © 2017 Klaile et al. 2017 Klaile et al. This content is distributed under the terms of the Creative Commons Attribution 4.0 International license.

*Candida albicans* (Berkhout; strains SC5314 and C28a/ATCC 10231) and *Candida glabrata* (Meyer and Yarrow; strain ATCC 2001) were kindly provided by Bernhard Hube (Leibniz Institute for Natural Product Research and Infection Biology—Hans Knöll Institute [HKI], Jena, Germany). If not mentioned otherwise, yeast cells of *C. albicans* SC5314 were used. Overnight cultures were inoculated with colonies from YPD plates and were grown in YPD medium at 30°C and 180 rpm. (YPD is 20 g/liter peptone, 10 g/liter yeast extract, and 20 g/liter glucose [pH 5.5 to 6], with 15 to 20 g/liter agar used for solid medium.) *C. albicans* germ tubes were induced in RPMI medium at 37°C for up to 2.5 h to a length of about 10 to 20 µm. For UV inactivation, 5 ml overnight culture was pelleted at 3,000 × *g*, washed with phosphate-buffered saline (PBS), and irradiated with 2 J/cm^2^ in 20 ml PBS in a 15-cm dish. For heat inactivation, cells were washed with PBS and incubated in 1 ml PBS at 65°C and 1,200 rpm in a heat block. Live and inactivated cells were washed twice with 20 ml PBS before experiments. In some cases, 2 × 10^8^ yeast cells were incubated with 7.5 µg proteinase K in 500 µl at 37°C for 1 h and washed extensively. For deglycosylation, 500 µl wet yeast pellet was solved in 1 ml PBS and incubated with approximately 3 mg of zymolyase (AMS Biotechnology, Abington, United Kingdom) for 1 h at room temperature and washed extensively.

### Recombinant proteins.

Recombinant proteins consisting of the respective CEACAM extracellular domain (human CEACAM1, CEACAM1dN lacking the N-terminal IgV-like domain, CEACAM3, CEACAM6, CEACAM8, and rat CEACAM1) fused to the constant region of human IgG were produced in HEK-293 cells and purified via protein G columns (GE Healthcare, Munich, Germany) as described previously (10, 21, 40). Recombinant human CEACAM5-Fc, human CEACAM7-His, and mouse CEACAM1-His were purchased from Sino Biological, Inc. (Beijing, People’s Republic of China), Abcam Plc (Cambridge, United Kingdom), and Hölzel Diagnostika GmbH (Cologne, Germany), respectively.

### Pulldown assays.

Pulldown assays were performed with 2 × 10^8^ live yeast cells or germ tubes from overnight cultures. Before the incubation with fungal cells, each recombinant protein was pretreated by the addition of at least 1 volume of 100 mM glycine (pH 2.2) in order to break up potential CEACAM homodimers. After 10 min, the pH was restored by the addition of 1 volume 1 M Tris-HCl (pH 8.0). Fungal cells were incubated for 2 h with the recombinant proteins, washed twice with PBS, and eluted with PBS–1% Triton X-100. Samples were analyzed by Western blotting for the presence of the respective recombinant protein.

### FITC-dextran translocation.

For the determination of paracellular FITC-dextran translocation, cells were grown in triplicate on 12-well Transwell inserts in complete, phenol red-free medium (upper chamber, 500 µl; lower chamber, 1,500 µl). FITC-dextran (4 kDa) was added at 0.5 mg to the upper chambers (apical side). Samples were collected from the lower chambers (basal side) after incubation for 24 h. FITC-dextran concentrations were determined after the addition of 1/10 volume Tris-HCl (pH 8.0) (excitation, 490 nm; emission, 520 nm; range, 0.016 to 200 µg/ml). Measurement was performed in a Tecan Infinite M200 microplate reader (Tecan Deutschland GmbH, Crailsheim, Germany).

### TEER.

Cells were grown on 12-well Transwell inserts in complete medium (upper chamber, 500 µl; lower chamber, 1,500 µl). (In some cases, phenol red-free complete medium was used.) TEER was measured using an EVOM2 epithelial volt ohmmeter (WPI [World Precision Instruments], Berlin, Germany). For the analysis of TEER values, blank filter resistance (100 Ω) was subtracted. All measurements were performed on a minimum of triplicate wells.

### Western blots.

Samples were run on 15 well Mini-Protean TGX gels (4 to 15%) under reducing conditions (5× reducing sample buffer, consisting of 14.5% SDS [wt/vol], 0.3 M Tris-HCl [pH 6.8], 50% glycerol, 0.015% bromphenol blue [wt/vol], and 20 mM dithiothreitol [DTT]). After protein transfer, nitrocellulose membranes were blocked with 10% milk for at least 1 h, and proteins were detected using the appropriate primary and HRP-conjugated secondary antibodies and SuperSignal West Pico chemiluminescent substrate (Fisher Scientific GmbH, Schwerte, Germany). Signals were detected using a Fusion FX7 imager (Peqlab Biotechnologie GmbH, Erlangen, Germany) and analyzed using the Image Studio Lite software (v5.2) and subsequent transformation in Microsoft Excel.

### Analysis of C2BBe1 cell culture supernatants, mucus fractions, and cell lysates.

Cells were grown in 6-well plates and stimulated in 2 ml complete medium for 72 h. (For subsequent ultracentrifugation analysis, phenol red-free complete medium was used.) After incubation, the cell culture supernatant (CCS) was removed carefully without disturbing the mucus layer. The cells were washed twice thoroughly with 1 ml prewarmed PBS (37°C) (Invitrogen, Darmstadt, Germany), and both wash fractions were collected together (mucus fraction). CCS and the mucus fraction were cleared from cellular debris by centrifugation at 16,000 × *g* for 10 min at 4°C. The respective supernatants were transferred into new tubes and either used directly for analysis (Western blotting and CXCL8 enzyme-linked immunosorbent assay [ELISA]) or subjected to ultracentrifugation. For ultracentrifugation analysis, PBS was added to the samples to a final volume of 5 ml, and samples were centrifuged at 100,000 × *g* for 90 min. Supernatants were concentrated in Amicon Ultra devices (Millipore Amicon Ultra 4-ml Ultracel-3 membrane, 3 kDa; Merck Chemicals GmbH, Darmstadt, Germany) in a swinging bucket rotor at 3,500 × *g* before Western blot analysis. Pellets were solved directly in 1× reducing sample buffer (see “Western blots” above).

Cells were homogenized in 300 µl ice-cold lysis buffer (20 mM HEPES [pH 7.4], 150 mM NaCl, 2 mM MgCl, 1% Triton X-100, 1 mM phenylmethylsulfonyl fluoride) supplemented with protease inhibitor cocktail (Roche cOmplete, EDTA-free protease inhibitor cocktail tablets; Sigma-Aldrich Chemie GmbH, Steinheim, Germany) and incubated on ice for 1 h. Samples were cleared by centrifugation at 16,000 × *g* for 10 min at 4°C and used for Western blot analysis.

### CXCL8 ELISA.

Supernatants were harvested at the indicated time points and tested for CXCL8 concentrations by ELISA (BD Biosciences, Heidelberg, Germany). As a CEACAM-independent positive control, cells were incubated with cytomix (25 ng/ml IL-1β, 50 ng/ml TNF-α, and 50 ng/ml IFN-γ).

### Phosphorylation analysis.

For phosphorylation assays, cells were grown in 10-cm dishes coated with collagen in PBS at a final concentration of 0.01% (collagen solution from bovine skin [0.3%]; Sigma-Aldrich Chemie GmbH, Steinheim, Germany). Since the total CEACAM1 expression was markedly reduced in 21-day-differentiated C2BBe1 cells and the short CEACAM1 isoforms (CEACAM1-4S and CEACAM1-3S) were expressed at higher levels than the long ITIM-bearing isoforms (compare the threshold cycle [*C*_*T*_] values in Fig. S1B), cells were allowed to differentiate for only 7 days. Cells were starved in 10 ml serum-free DMEM for at least 2 h and stimulated by the addition of 10^9^ live *C. albicans* yeast cells (multiplicity of infection [MOI] of 100) for the indicated times. Cell culture supernatants were removed, and cells were lysed in 1 ml ice-cold lysis buffer (see above) supplemented with PhosSTOP phosphatase inhibitor cocktail (Roche Diagnostics Deutschland GmbH, Mannheim, Germany). After incubation on ice for 1 h, samples were cleared by centrifugation at 16,000 × *g* for 10 min at 4°C and subjected to immunoprecipitation using 2 µg anti-human CEACAM1 antibody (B3-17) preincubated with 20 µl protein G Sepharose 4 Fast Flow (GE Healthcare, Munich, Germany). Samples were incubated on a rotor at 4°C for 3 to 5 h and washed three times with lysis buffer. The supernatants were removed completely, and 20 µl 1× reducing sample buffer (see above) was added to the Sepharose pellet. Samples were heated to 95°C for 5 min at 1300 rpm and analyzed by Western blotting for the presence of phosphotyrosine (4G10 antibody). Membranes were redeveloped for CEACAM1 using the polyclonal pan-CEACAM antibody.

### Adhesion assays.

A total of 10^4^ *C. albicans* yeast cells per well (48-well plates) were incubated with confluent HeLa cells for 60 min and confluent C2BBe1 cells (differentiated for 3 days and washed twice with warm medium to remove mucus from the cell monolayer) for 30 min. In some cases, *C. albicans* yeast cells were preincubated for 2 h with cell culture supernatants of C2BBe1 cells left untreated or treated with CaUV for 72 h and used for adhesion in the presence of the respective preconditioned media. After being washed three times with warm PBS, adherent yeast cells were plated and colonies were counted after 24 to 48 h.

### Statistics.

Data are presented as single data points: the respective means ± standard deviations (SD) are indicated by wide and narrow bars (if applicable). A minimum of three independent experiments were performed and analyzed for each data set presented. Paired or unpaired two-sided *t* tests (as indicated) were performed using GraphPad Prism version 5.04 for Windows (GraphPad Software, Inc., San Diego, CA). Due to differences in the total intensities between individual Western blots, relative intensity units given by the Image Studio Light Software (v5.2) from the quantification of Western blot bands were analyzed by two-sided paired *t* test. *P* < 0.05 was considered statistically significant.
