# Supplementary figures and images for: Binding of Candida albicans to Human CEACAM1 and CEACAM6 Modulates the Inflammatory Response of Intestinal Epithelial Cells
Source: mBio. 2017 Mar 14;8(2):e02142-16. doi: 10.1128/mBio.02142-16 (PMC5350469; doi:10.1128/mBio.02142-16)

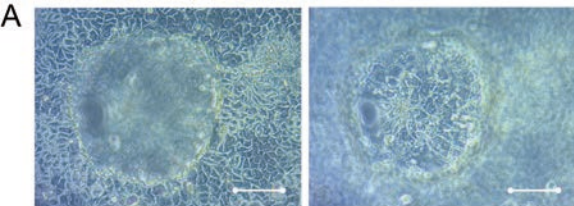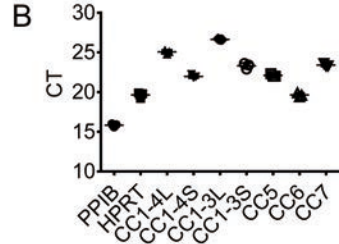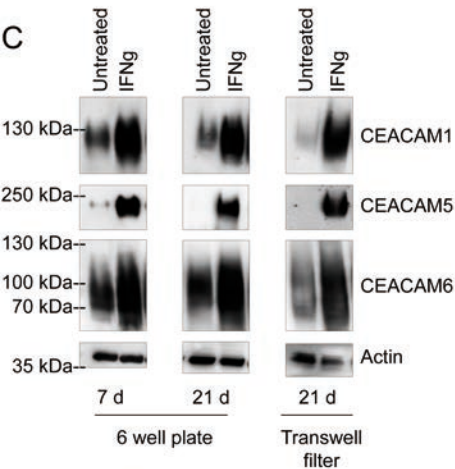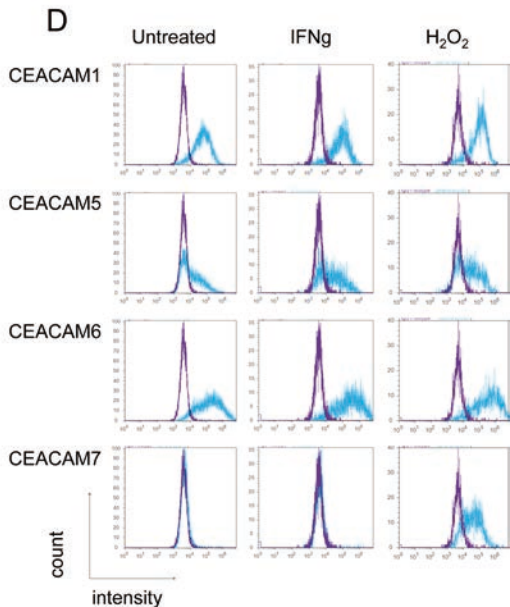

Supplement: FIG S1 [file mbo002173234sf1.pdf]

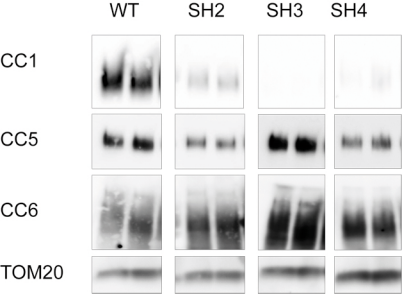

Supplement: FIG S2 [file mbo002173234sf2.pdf]

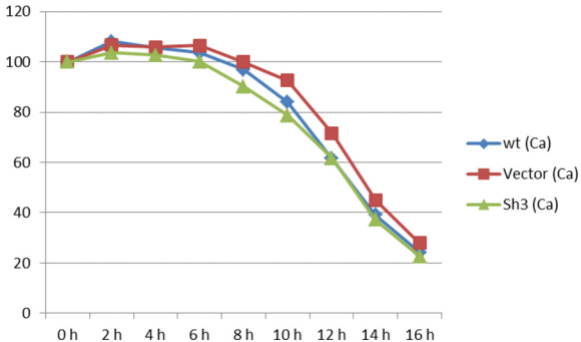

Supplement: FIG S3 [file mbo002173234sf3.pdf]

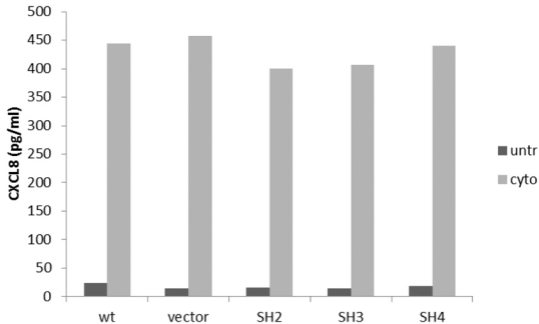

Supplement: FIG S4 [file mbo002173234sf4.pdf]

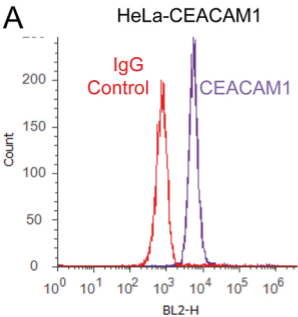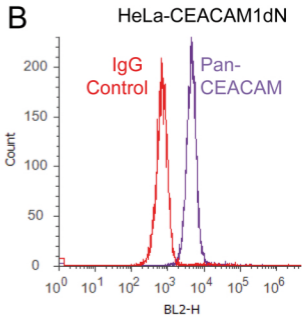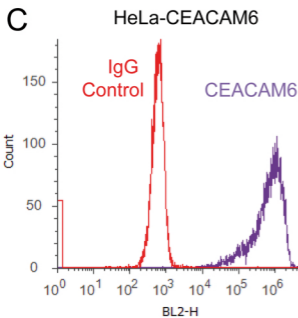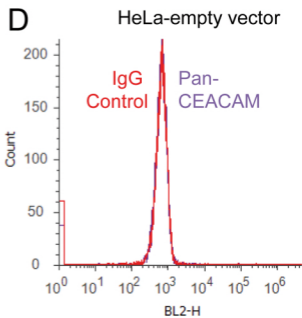

Supplement: FIG S6 [file mbo002173234sf6.pdf]
