# Supplementary material for: Binding of Candida albicans to Human CEACAM1 and CEACAM6 Modulates the Inflammatory Response of Intestinal Epithelial Cells
Source: mBio. 2017 Mar 14;8(2):e02142-16. doi: 10.1128/mBio.02142-16 (PMC5350469; doi:10.1128/mBio.02142-16)
Supplement: FIG S5 [file mbo002173234sf5.pdf]

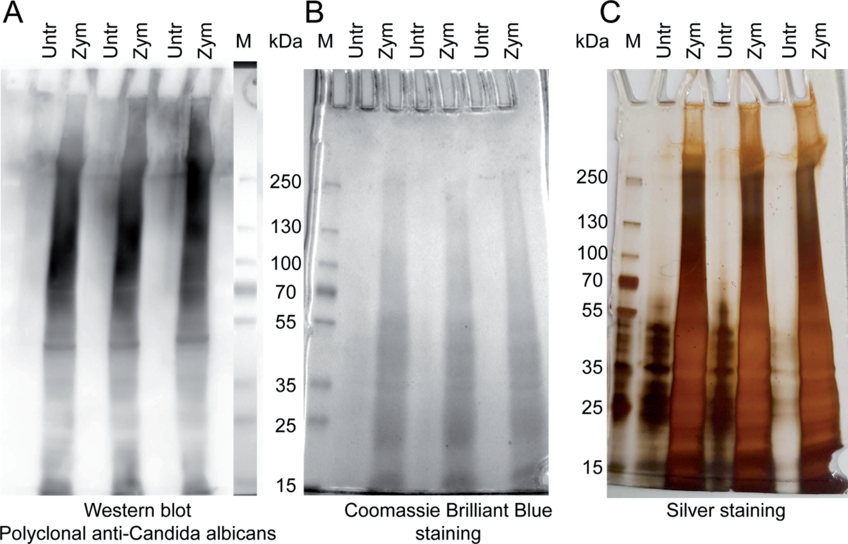

A
Untr
Zym
Untr
Zym
Untr
Zym
M
Western blot
Polyclonal anti-Candida albicans
B
kDa
M
Untr
Zym
Untr
Zym
Untr
Zym
250
130
100
70
55
35
25
15
Coomassie Brilliant Blue staining
C
kDa
M
Untr
Zym
Untr
Zym
Untr
Zym
250
130
100
70
55
35
25
15
Silver staining
